# Y chromosome microdeletions in Chinese men with infertility: prevalence, phenotypes, and intracytoplasmic sperm injection outcomes

**DOI:** 10.1186/s12958-023-01168-5

**Published:** 2023-12-05

**Authors:** Dongjia Chen, Guoqing Fan, Xianqing Zhu, Qinyun Chen, Xuren Chen, Feng Gao, Zexin Guo, Peng Luo, Yong Gao

**Affiliations:** Reproductive Medicine Center, Guangdong Provincial Key Laboratory of Reproductive Medicine, Guangdong Provincial Clinical Research Center for obstetrical and gynecological diseases, The First Affiliated Hospital, Sun Yat-sen University, 510080, Guangzhou, China

**Keywords:** Y chromosome microdeletion, AZF, Azoospermia, Cryptozoospermia, Oligozoospermia, Intracytoplasmic sperm injection

## Abstract

**Background:**

The incidence of Y chromosome microdeletions varies among men with infertility across regions and ethnicities worldwide. However, comprehensive epidemiological studies on Y chromosome microdeletions in Chinese men with infertility are lacking. We aimed to investigate Y chromosome microdeletions prevalence among Chinese men with infertility and its correlation with intracytoplasmic sperm injection (ICSI) outcomes.

**Methods:**

This single-center retrospective study included 4,714 men with infertility who were evaluated at the Reproductive Center of the First Affiliated Hospital of Sun Yat-sen University between May 2017 and January 2021. Semen analysis and Y-chromosome microdeletion via multiplex polymerase chain reaction were conducted on the men. The study compared outcomes of 36 ICSI cycles from couples with male azoospermia factor (AZF)cd deletions with those of a control group, which included 72 ICSI cycles from couples without male Y chromosome microdeletions, during the same period. Both groups underwent ICSI treatment using ejaculated sperm.

**Results:**

Among 4,714 Chinese men with infertility, 3.31% had Y chromosome microdeletions. The combined deletion of sY254 and sY255 in the AZFc region and sY152 in the AZFd region was the prevalent pattern of Y chromosome microdeletion, with 3.05% detection rate. The detection rates of AZF deletions in patients with normal total sperm count, mild oligozoospermia, severe oligozoospermia, cryptozoospermia, and azoospermia were 0.17%, 1.13%, 5.53%, 71.43%, and 7.54%, respectively. Compared with the control group, the AZFcd deletion group exhibited no significant difference in the laboratory results or pregnancy outcomes of ICSI cycles using ejaculated sperm.

**Conclusions:**

This is the largest epidemiological study on Y chromosome microdeletions in Chinese men with infertility. The study results underline the necessity for detecting Y chromosome microdeletion in men with infertility and severe sperm count abnormalities, especially those with cryptozoospermia. The combined deletion of sY254 and sY255 in the AZFc region and sY152 in the AZFd region was the most prevalent Y chromosome microdeletion pattern. Among patients with AZFcd deletion and ejaculated sperm, ICSI treatment can result in pregnancy outcomes, similar to those without AZFcd deletion.

**Supplementary Information:**

The online version contains supplementary material available at 10.1186/s12958-023-01168-5.

## Background

**Fig. 1:**
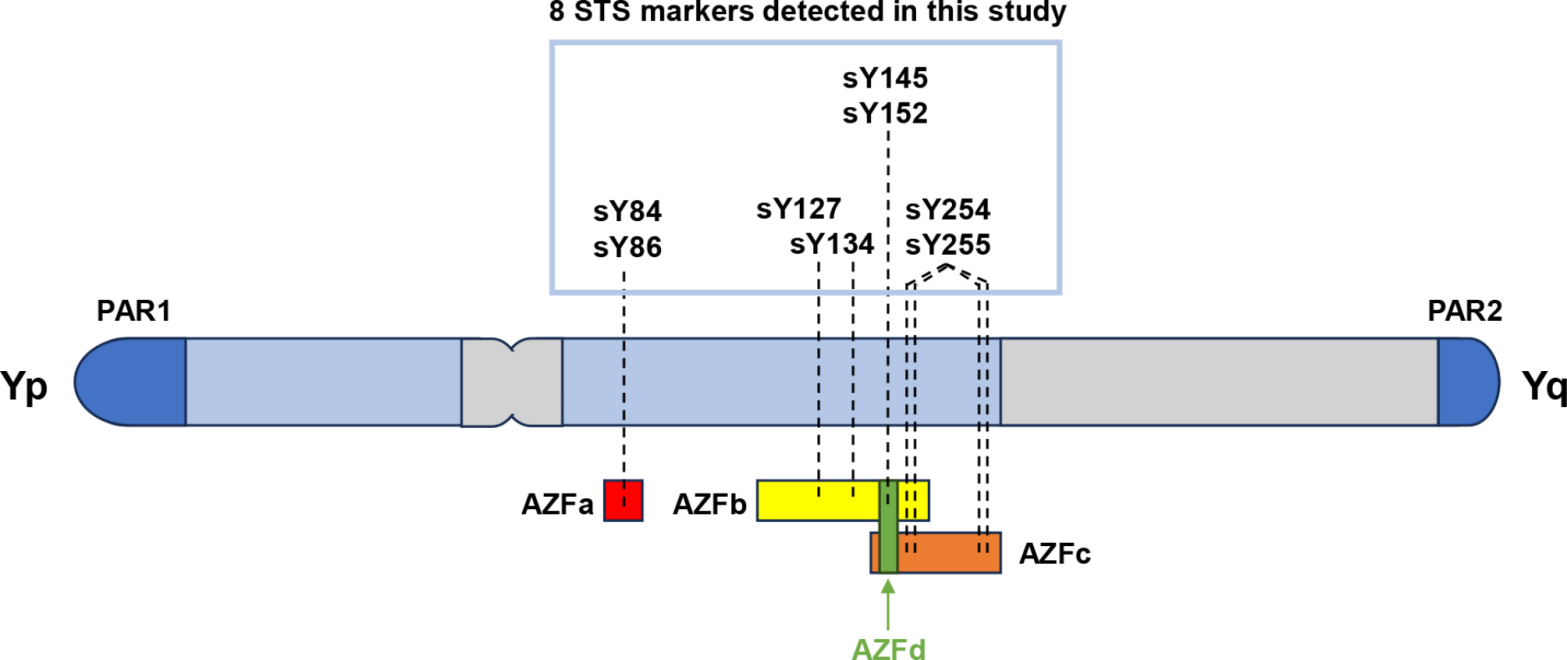
Diagram of the human Y chromosome showing the AZF loci and associated 8 STS markers detected in this study Y chromosome microdeletion involves the complete or partial deletion of the AZF on the long arm of the Y-chromosome at the subchromosome level. The AZF region can be divided into AZFa, AZFb, and AZFc, from the proximal to the distal end in the Y chromosome. A possible AZFd region has been proposed to exist between AZFb and AZFc. The associated 8 STS markers detected in this study are as follows: AZFa (sY84 and sY86), AZFb (sY127 and sY134), AZFc (sY254 and sY255), and AZFd (sY145 and sY152)

Infertility affects approximately 15% of couples of childbearing ages worldwide, with 20–70% being caused by male-independent factors [1]. Male infertility is associated with various factors. Decreased sperm count is a key risk factor for male infertility [2]. Treating male infertility caused by severe oligozoospermia and azoospermia is challenging. Genetic factors play an important role in the etiology of spermatogenic disorders, with Y chromosome microdeletions being a common genetic factor. Y chromosome microdeletion involves the complete or partial deletion of the azoospermia factor (AZF) on the long arm of the Y-chromosome at the subchromosome level. The AZF region can be divided into AZFa, AZFb, and AZFc, from the proximal to the distal end in the Y chromosome [3]. A possible AZFd region has been proposed to exist between AZFb and AZFc, but its clinical significance is still a matter of debate (Fig. 1) [4]. The AZF region contains important genes such as *USP9Y*, *RBM*, and *DAZ* that are involved in spermatogenesis and testicular development [5]. Therefore, the clinical outcome of lacking different areas of AZF manifests as different degrees of reduced sperm count, varying from oligozoospermia to azoospermia.

The incidence of Y chromosome microdeletions varies among men with infertility across regions and ethnicities worldwide. This variation, ranging from 3.3 to 10.9% is primarily due to differences in patient selection criteria and sample size in various regions and races [6–10]. Comprehensive epidemiological studies on Y chromosome microdeletions in Chinese men with infertility are lacking. Therefore, large-scale epidemiological studies are needed to understand the prevalence of Y chromosome microdeletions in populations with different sperm counts and establish indications for Y chromosome microdeletion testing.

Most patients with AZFa/b region microdeletions exhibit azoospermia, whereas patients with AZFc region microdeletions exhibit variable clinical and histological phenotypes [3]. Men with AZFc region microdeletions and severe oligozoospermia may achieve offspring development via intracytoplasmic sperm injection (ICSI) [2, 3]. Most studies demonstrate that ICSI outcomes of patients with AZFc microdeletions resemble those of controls without Y chromosome microdeletion [11–15]. However, some studies report poor ICSI outcomes for patients with AZFc microdeletions [16–18]. Owing to the conflicting results of the aforementioned studies, additional confirmation is required to ascertain the effect of Y chromosome microdeletions on ICSI outcomes.

Therefore, we aimed to conduct an epidemiological study on Y-chromosome microdeletions involving 4,714 Chinese men with infertility to provide additional evidence for clinical diagnosis, assisted reproductive technology treatment, and genetic counseling.

## Materials and methods

### Patients

We retrospectively included 4,714 infertile men who underwent reproductive evaluation at the Reproductive Center of the First Affiliated Hospital of Sun Yat-sen University between May 2017 and January 2021 and obtained semen and Y chromosome microdeletion examination reports. All infertile men included had abnormal semen parameters, including sperm count, motility, or morphology.

We included 36 ICSI cycles involving patients with AZFcd microdeletions to explore the effect of Y chromosome microdeletions on ICSI prognosis. Among these patients, 3 had mild oligozoospermia, 13 had severe oligozoospermia, and 20 had cryptozoospermia. The chromosome number and structure of these men were normal, except for the sY254, sY255, and sY152 deletions in the AZFcd region.

Additionally, we selected 72 ICSI cycles from men with infertility and oligozoospermia or cryptozoospermia without AZF microdeletion in the same period as the control group, using propensity score matching at a 1:2 ratio. Matching parameters included male and female age, infertility duration, female body mass index (BMI), number of previous deliveries, retrieved oocyte count, and mature oocyte count (metaphase II oocytes).

All the women included in the ICSI cycle had normal karyotypes. Couples with uterine malformations, endometriosis, polycystic ovary syndrome, family history of genetic disease, and other infertility factors potentially influencing oocyte quality and embryo implantation were excluded.

### Semen examination

The men with infertility provided semen samples for examination through masturbation after 2–7 d of abstinence. Semen was collected in a semen collection cup and analyzed for sperm count and motility within 1 h using an automatic sperm analyzer. When no sperm were detected, the sample was centrifuged at 3000 *g* for 15 min and manually searched for sperm in the centrifuge precipitation. Semen smears and staining were performed to evaluate sperm morphology.

### Y chromosome microdeletion analysis

Genomic deoxyribonucleic acid (gDNA) was extracted from the patients’ peripheral venous blood cells and analyzed using the human Y chromosome AZF microdeletion nucleic acid test kit (IPE BIOTECHNOLOGY, Beijing) to detect potential microdeletions in the Y chromosome AZF region. The kit targets 8 STS loci in the Y chromosome AZF region as the target sequence, and employs specific primers, fluorescent probes, heat-resistant DNA polymerase (Taq enzyme), and four types of nucleotide monomers (dNTPs). Real-time fluorescent quantitative polymerase chain reaction (PCR) was conducted on each sample, involving four triple PCR reactions, with each reaction system containing an internal control and specific primers and probes for two loci. The primers and probes used for the detection of STS markers in this study were designed based on the EAA/EMQN best practice guidelines [3] and recent studies [19, 20]. This allowed for the rapid detection of potential deletions at the 8 loci within the Y chromosome AZF region. The different regions of AZF and corresponding STS markers are as follows: AZFa (sY84 and sY86), AZFb (sY127 and sY134), AZFc (sY254 and sY255), and AZFd (sY145 and sY152). The composition of the four reactions included: reaction 1 detecting β-actin, sY84, sY86; reaction 2 detecting SRY, sY127, sY152; reaction 3 detecting SRY, sY254, sY134; reaction 4 detecting SRY, sY145, sY255. The ABI 7500 fluorescent quantitative PCR instrument was used for detection, with fluorescent detection channels including FAM, VIC, and CY5, and reaction conditions set according to the instruction manual.

β-actin and SRY were used as internal controls. The quality control process involved: (1) Negative control samples (female gDNA) showing an S-shaped amplification curve with a Ct value ≤ 26 in reaction 1’s Cy5 channel (β-actin), while all other channels had Ct values > 28 or no Ct value displayed. (2) Positive control samples (normal male gDNA) showing S-shaped amplification curves with Ct values ≤ 26 in all channels for the four reactions. If the above conditions were not simultaneously met, the test was considered invalid, and all tests were repeated. (3) The test sample’s reaction 1 in the Cy5 channel (β-actin) should exhibit an S-shaped amplification curve with a Ct value ≤ 28. Samples not meeting condition 3 were reprocessed and retested before evaluation.

### Standards for distinguishing different sperm count phenotypes

Oligozoospermia is considered when the total sperm counts per ejaculate is < 39 × 10^6^. Oligozoospermia is further classified as mild oligozoospermia when total sperm per ejaculate is ≥ 10 × 10^6^ and severe oligozoospermia when the total sperm per ejaculate is < 10 × 10^6^. Cryptozoospermia refers to absent spermatozoa in fresh preparations, but visible after centrifuging semen at 3000 *g* for 15 min. Azoospermia indicates no sperm detected in the direct microscopic examination of semen and after centrifuging the semen.

### ICSI procedures

All women were treated with a gonadotropin-releasing hormone agonist or antagonist regimen to control ovarian stimulation. When the dominant follicle monitored via ultrasound reached ≥ 18 mm in diameter, human chorionic gonadotropin was injected intramuscularly. Ultrasound-guided ovarian puncture was conducted 36 h later, followed by ICSI using ejaculated sperm. Based on the patient and embryos, 1–2 high-quality embryos were selected for intrauterine transfer under abdominal ultrasound guidance. After embryo transfer, women received conventional corpus luteum support treatment. Excess high-quality embryos were frozen based on the couple’s preferences, thawed, and transferred to the subsequent natural or artificial hormone replacement cycle.

### Outcome calculation

The couples’ ages were calculated from the time of entering the ICSI cycles. Normal fertilization rate was defined as the percentage obtained by dividing the number of two pronuclei (2PN) embryos by the number of metaphase II oocytes. The high-quality embryo rate was calculated by dividing the number of high-quality embryos by the number of 2PN embryos. The blastocyst formation rate was calculated by dividing the number of blastocysts formed by that of cultured blastocysts. The high-quality blastocyst rate represents the percentage of high-quality blastocysts divided by the number of blastocysts formed. The cumulative clinical pregnancy rate was the percentage of clinical pregnancies divided by the total oocyte-retrieval cycles. The cumulative live birth rate was calculated as the percentage of live birth cycles divided by the number of oocyte retrieval cycles. The cumulative miscarriage rate was calculated from the number of clinical pregnancies that did not result in live births.

### Statistical analysis

IBM SPSS Statistics for Windows (version 22.0 [IBM Corp., Chicago, IL, USA]) was used for the data analysis. Measurement data were presented as mean ± standard deviation (x ± s), and groups were compared using student’s t-test or Mann–Whitney U test. Counting data were represented as number of cases (percentage; n [%]), and group comparisons were conducted using Pearson’s χ ^2^ or Fisher’s exact test. The ICSI cycles of the AZFcd deletion and control groups were matched using the propensity score matching method at a 1:2 ratio with a 0.2 caliper value.

### Ethics Statement

The ethics committee of the First Affiliated Hospital of Sun Yat-sen University approved the study protocol (Approval number: [2021]398; Date of approval: June 3, 2021). Owing to the retrospective nature of this study, the requirement for informed consent was waived.

## Results

### Incidence of Y chromosome microdeletions

Of the 4,714 men with infertility, 2,412(51.17%) had normal total sperm numbers, 703(14.91%) had mild oligozoospermia, 615(13.05%) had severe oligozoospermia, 56(1.19%) had cryptozoospermia, and 928(19.69%) had azoospermia. The detection rate of Y chromosome microdeletions in the men with infertility population was 3.31%(156/4714). Table 1 presents the detection rates of Y chromosome microdeletions across sperm count phenotypes. The AZF deletion detection rates in men with normal total sperm numbers was 0.17%(4/2712). The AZF deletion detection rates in patients with mild oligozoospermia, severe oligozoospermia, cryptozoospermia, and azoospermia were 1.13% (8/703), 5.53% (34/615), 71.43% (40/56), and 7.54% (70/928), respectively. Additional file 1 presents the detailed results of semen analysis and Y-chromosome microdeletion analysis of the 4,714 men with infertility.

**Table 1:** Detection rate of Y chromosome microdeletion in different sperm count phenotype

Group	Normal	Mild oligozoospermia	Severe oligozoospermia	Cryptozoospermia	Azoospermia
Number of patients	2412	703	615	56	928
Number of patients with AZF deletion	4^a^	8^b^	34^c^	40^d^	70^e^
Detection rate, %^f^	0.17	1.13	5.53	71.43	7.54

^a^Two patients had sY86 deletions, and two patients had sY254, sY255, and sY152 deletions

^b^One patient had an sY86 deletion, and seven patients had sY254, sY255, and sY152 deletions

^c^34 patients had sY254, sY255, and sY152 deletions

^d^40 patients had sY254, sY255, and sY152 deletions

^e^24 patients had sY254, sY255, and sY152 deletions, one patient had sY84 and sY86 deletions, eight patients had sY127 and sY134 deletions, three patients had sY254, sY255, sY145, and sY152 deletions, eight patients had sY127, sY134, sY254, sY255, and sY152 deletions, 22 patients had sY127, sY134, sY254, sY255, sY145, and sY152 deletions, and four patients had all eight STS deletions

^f^The detection rates between the severe oligozoospermia and azoospermia groups showed no significant difference (*P* = 0.122). However, significant differences were observed in the pairwise comparisons between the other groups (*P* < 0.05)

Abbreviations: AZF, azoospermia factor

Table 2 presents the number and frequency of deletions at each STS site. The detection rates of sY254, sY255, and sY152 deletions were the same and highest at 3.05%(144/4714), as isolated sY152 deletion did not exist. Moreover, sY152 deletion was consistently co-deleted with sY254 and sY255 deletions.

**Table 2:** Deletion of STS in the 4,714 men with infertility

AZF	AZFa		AZFb		AZFc		AZFd	
STS	sY84	sY86	sY127	sY134	sY254	sY255	sY145	sY152
Number of STS Deletions	5	8	42	42	144	144	29	144
Frequency	0.11%	0.17%	0.89%	0.89%	3.05%	3.05%	0.62%	3.05%

Abbreviations: AZF, azoospermia factor; STS, sequence-tagged site

Table 3 presents the STS deletion patterns and corresponding sperm count phenotypes of the 156 men with infertility and Y chromosome microdeletions. Six microdeletion patterns only corresponded to azoospermia, including AZFa complete deletion (0.64%, 1/156), AZFb complete deletion (5.13%, 8/156), AZFcd complete deletion (1.92%, 3/156), AZFbcd complete deletion (14.1%, 22/156), AZFabcd complete deletion (2.56%, 4/156), and AZFbc complete deletion combined with AZFd region sY152 deletion (5.13%, 8/156). The most prevalent microdeletion pattern was the combination of sY254 and sY255 deletions in the AZFc region and sY152 deletions in the AZFd region (68.59%, 107/156), corresponding to a rich sperm count polymorphism, including normal, oligozoospermia, cryptozoospermia, and azoospermia. We did not identify any patient with only complete AZFd or AZFc deletion.

**Table 3:** Pattern of STS deletions in 156 men with infertility and Y chromosome microdeletion and corresponding sperm count phenotype

Number of patients (%)	AZFa		AZFb		AZFc		AZFd		Sperm count phenotype
	sY84	sY86	sY127	sY134	sY254	sY255	sY145	sY152	
1(0.64%)	-	-	+	+	+	+	+	+	Azoospermia
3(1.92%)	+	-	+	+	+	+	+	+	Normal, mild oligozoospermia^a^
8(5.13%)	+	+	-	-	+	+	+	+	Azoospermia
107(68.59%)	+	+	+	+	-	-	+	-	Normal, oligozoospermia, cryptozoospermia, azoospermia^b^
3(1.92%)	+	+	+	+	-	-	-	-	Azoospermia
8(5.13%)	+	+	-	-	-	-	+	-	Azoospermia
22(14.1%)	+	+	-	-	-	-	-	-	Azoospermia
4(2.56%)	-	-	-	-	-	-	-	-	Azoospermia

+: STS exists; -: STS was deleted

^a^Two patients had normal total sperm count and one had mild oligozoospermia

^b^Two patients had normal total sperm count, seven had mild oligozoospermia, 34 had severe oligozoospermia, 40 had cryptozoospermia, and 24 had azoospermia

**Fig. 2:**
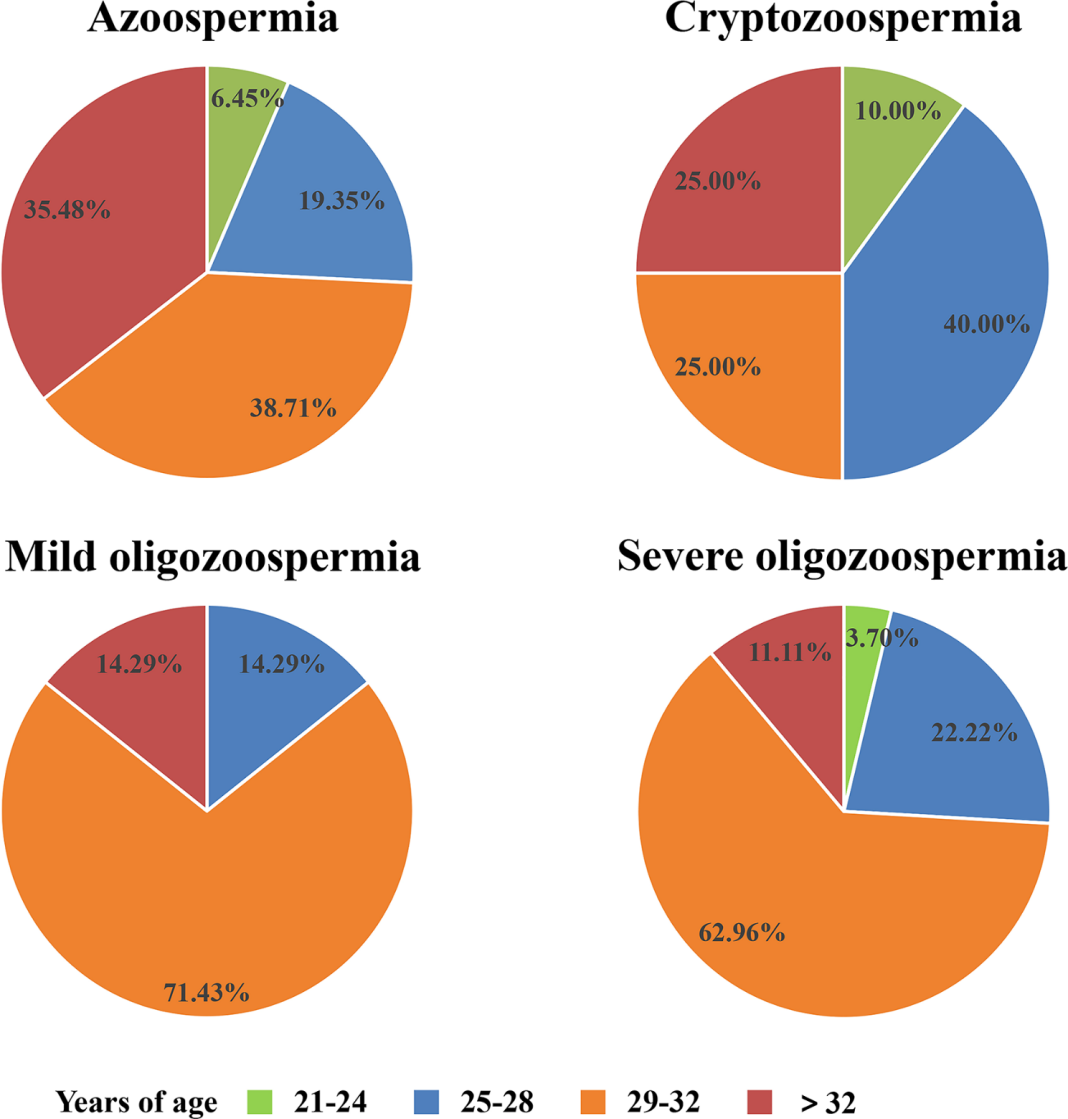
The proportion of patients with AZFcd (sY254, sY255, and sY152) deletion in different age stratified by azoospermia, cryptozoospermia, mild oligozoospermia and severe oligozoospermia. Patients above 29 years old had the highest proportion among patients with AZFcd deletion having azoospermia, oligozoospermia, and cryptozoospermia

Figure 2 shows the proportion of patients with AZFcd deletion (combination of sY254, sY255, and sY152 deletions) at different ages, stratified based on azoospermia, cryptozoospermia, mild oligozoospermia, and severe oligozoospermia. We observed that among patients with azoospermia, oligozoospermia, and cryptozoospermia the proportion above 29 years of age was the highest.

### ICSI outcomes

Table 4 presents the basic characteristics and ICSI outcomes of the AZFcd deletion (combination of sY254, sY255, and sY152 deletions) and control groups. No significant differences were observed in the men and women ages, infertility duration, female BMI, number of previous deliveries, retrieved oocyte count, or mature oocyte count.

**Table 4:** Clinical characteristics and ICSI outcomes of AZFcd deletion (combination of sY254, sY255, and sY152 deletions) and control group

	AZFcd deletion group (*n* = 36)	Control group (*n* = 72)	*P*-value
**Baseline characteristics**			
Male age, y	31.67 ± 4.74	31.00 ± 4.20	0.458
Female age, y	29.53 ± 3.91	29.21 ± 3.39	0.662
Women’s BMI,	20.25 ± 1.76	20.50 ± 1.97	0.510
Duration of infertility	3.67 ± 2.72	3.61 ± 2.20	0.909
Number of births in females before	0.06 ± 0.23	0.01 ± 0.12	0.317
Number of oocytes retrieved	12.61 ± 5.76	12.31 ± 5.83	0.797
Number of MII oocytes retrieved	10.44 ± 5.19	10.21 ± 5.07	0.821
**ICSI outcomes**			
Normal fertilization rate,%	71.85 ± 14.45	78.11 ± 19.74	0.095
High-quality embryo rate,%	42.66 ± 25.45	49.13 ± 26.23	0.222
Blastocyst formation rate,%	62.55 ± 37.47	53.07 ± 34.97	0.314
High-quality blastocyst rate,%	60.13 ± 43.48	49.03 ± 36.74	0.329
Total number of ET cycles	54	89	/
Number of transferred embryos per ET	1.80 ± 0.53	1.87 ± 0.43	0.421
Blastocyst transfer cycles, *n*(%)	17/54(31.48)	33/89(37.08)	0.496
FET cycles, *n*(%)	18/54(33.33)	40/89(44.94)	0.170
Clinical pregnancy rate per fresh ET, *n*(%)	19/36(52.78)	26/49(53.06)	0.979
Live birth rate per fresh ET, *n*(%)	15/36(41.67)	23/49(46.94)	0.629
Miscarriage rate per fresh ET, *n*(%)	4/19(21.05)	3/26(11.54)	0.650
Clinical pregnancy rate per FET, *n*(%)	6/18(33.33)	20/40(50.00)	0.238
Live birth rate per FET, *n*(%)	5/18(27.78)	18/40(45.00)	0.215
Miscarriage rate per FET, *n*(%)	1/6(16.67)	2/20(10.00)	0.779
Cumulative clinical pregnancy rate, *n*(%)	25/36(69.44)	46/72(63.89)	0.566
Cumulative live birth rate, *n*(%)	20/36(55.56)	41/72(56.94)	0.891
Cumulative miscarriage rate, *n*(%)	5/25(20.00)	5/46(10.87)	0.484

*Values are mean ± SD (95% CI)

Abbreviations: OPU, ovum pick-up; AZF, azoospermia factor; BMI, body mass index; ICSI, intracytoplasmic sperm injection; ET, embryo transfer; FET, frozen-thawed embryo transfer; CI, confidence interval; SD, standard deviation

The laboratory results revealed that the two groups had similar normal fertilization rate (71.85 ± 14.45% vs. 78.11 ± 19.74%, *P* = 0.095), high-quality day 3 embryo rate (42.66 ± 25.45% vs. 49.13 ± 26.23%, *P* = 0.222), blastocyst formation rate (62.55 ± 37.47% vs. 53.07 ± 34.97%, *P* = 0.314), and high-quality blastocyst rate (60.13 ± 43.48% vs. 49.03 ± 36.74%, *P* = 0.329).

The two groups exhibited no significant difference in the proportion of fresh embryo and thawed embryo transfer cycles (*P* = 0.170) or the proportion of day 3 embryo and blastocyst transfer cycles (*P* = 0.496). The number of embryos transferred per embryo transfer cycle was statistically comparable between both groups. The cumulative clinical pregnancy rate (69.44% vs. 63.89%, *P* = 0.566), cumulative live birth rate (55.56% vs. 56.94, *P* = 0.891), and cumulative miscarriage rate (20.00% vs. 10.87%, *P* = 0.484) in the AZFcd deletion group were similar to those in the control group.

## Discussion

This study, based on a large cohort of men with infertility in China, investigated the incidence of Y chromosome microdeletions and ICSI outcomes in patients with AZFcd deletions. Our results underline the necessity for detecting Y chromosome microdeletions in men with infertility and severe sperm count abnormalities, especially those with cryptozoospermia. The co-deletion of sY254 and sY255 in AZFc and sY152 in AZFd was the most prevalent pattern of Y chromosome microdeletion observed. Patients with AZFcd deletions (combination of sY254, sY255, and sY152 deletions) using ejaculated sperm and without Y chromosome microdeletions achieved similar ICSI outcomes.

Several studies have reported the incidence of Y chromosome microdeletions in Chinese populations; however, they only enrolled men with oligozoospermia and azoospermia [8, 10, 21, 22]. To our knowledge, this study is the largest epidemiological investigation of Y chromosome microdeletions and the first to report their incidence (3.13%) in Chinese men with infertility. One novel finding is that the Y chromosome microdeletions incidence among Chinese men with infertility with normal sperm counts is 0.17%, lower than the 2% incidence reported in normal men by the American Society of Reproductive Medicine Practice [23].

We detected AZF deletion rates in patients with mild oligozoospermia, severe oligozoospermia, and azoospermia of 1.13% (8/703), 5.53% (34/615), and 7.54% (70/928), respectively (slightly lower than the reported incidence of Y chromosome microdeletion in oligozoospermia [3.87–13.2%] and azoospermia [11.49–17.8%] in Chinese men with infertility, where oligozoospermia was defined based on sperm concentration) [8, 10, 21, 22]. However, the fifth edition of the World Health Organization laboratory manual for the examination and processing of human semen emphasizes that the total sperm number per ejaculate has a higher diagnostic value than sperm concentration [24]. Therefore, we utilized total sperm number to define oligozoospermia (total sperm number < 39 × 10^6^ per ejaculate).

This study identified variations in the incidence of Y chromosome microdeletions in patients with different phenotypes of total sperm number. The incidence of Y chromosome microdeletions in patients with cryptozoospermia was 71.43%, higher than that reported in previous studies (5–11.5%) [25, 26]. This study has two implications. First, screening for Y chromosome microdeletions among patients with cryptozoospermia may be cost-effective. Second, the sperm-discovering method through semen centrifugation should be regarded, as it can help patients with suspected azoospermia determine whether they have cryptozoospermia, which has a high probability of detecting a Y chromosome microdeletion. We observed a high detection rate of Y chromosome microdeletions in Chinese men with infertility having severe oligozoospermia (total sperm number < 10 × 10^6^ per ejaculate), cryptozoospermia, and azoospermia, suggesting that Y chromosome microdeletion testing should be considered when the total sperm number is below 10 × 10^6^ per ejaculate.

Kent-First et al. first proposed the AZFd region in 1999, situating it between the AZFb and AZFc regions (Fig. 1) [4]. However, the AZFd region’s clinical implications remain unclear. The EAA/EMQN best practice guidelines for the molecular diagnosis of Y chromosomal microdeletions do not recommend testing AZFd regions, as the sequence of the male-specific Y chromosome region and the mechanism underlying these microdeletions indicate its nonexistence [3]. However, the AZFd region could be related in spermatogenesis and recurrent pregnancy loss [27, 28]. The guidelines and expert consensus for diagnosing and treating andrological diseases in China recommend testing AZFd regions (sY145 and sY152) [29]. Therefore, we explored the AZFd region deletion incidence and its relationship with the total sperm count phenotype. We observed that all AZFc deletions were accompanied by SY152 deletion, the predominant deletion pattern in Chinese individuals with infertility, and that SY145 in AZFd may be associated with azoospermia. When sY145 was present, the semen parameters of the AZFcd deletion group were polymorphic, ranging from normal to oligozoospermia, cryptozoospermia, and azoospermia. However, when sY145 was deleted, the patients had only azoospermia, hence the importance of detecting SY145, a gene locus, as it may contain important genes associated with spermatogenesis. More case studies are needed to confirm our AZFd regions’ observations.

Compared with patients without Y chromosome microdeletion, those with AZFc deletions can achieve similar ICSI outcomes using ejaculated sperm, but worse ICSI outcomes using testicular sperm [12, 17, 30, 31]. Studies exploring ICSI outcomes in patients with combined deletions of STS sites in the AZFc and AZFd regions are lacking. Our study suggests that patients with AZFcd deletions (combination of sY254, sY255, and sY152 deletions) using ejaculated sperm could achieve ICSI outcomes similar to those in patients without Y chromosome microdeletions, consistent with most previous studies involving patients with AZFc deletions [12, 31]. Basic data such as female BMI, female hormones, and polycystic ovary syndrome in the ovulation cycle couples to match the control group was used, reducing potential bias. Unfortunately, we did not investigate the ICSI outcome using testicular sperm in patients with AZFcd deletions owing to the small number of patients who underwent testicular sperm extraction surgery.

Usually, the relationship between age and male semen parameters change (total sperm number and concentration) after 34 years of age [32, 33]. Stone et al. analyzed semen data from 5,081 men aged 16.5–72.3 years and observed a decrease in the total sperm number after 34 years of age [32]. In a study involving 71,623 Chinese men with infertility, the critical age for the decrease in total sperm count was 42 years [33]. However, the variation pattern of total sperm count with age in patients with AZFcd deletions remains unclear. Among patients with azoospermia, oligozoospermia, cryptozoospermia and AZFcd deletion, those over 29 years had the highest proportion. Our study could not conclusively determine whether patients with AZFcd region deletion experience an earlier decline in total sperm number than those without Y chromosome microdeletion owing to the limited number of patients with AZFcd deletion (107 patients). Thus, future studies with larger sample sizes are required.

This study had several advantages. First, this is the largest epidemiological study on Y chromosome microdeletions in Chinese men with infertility, presenting the incidence of Y chromosome microdeletion in Chinese population with normal sperm count (0.17%) for the first time. Second, we discovered that the most prevalent pattern of Y chromosome microdeletion is the co-deletion of sY254 and sY255 in AZFc and sY152 in AZFd in Chinese men with infertility (68.59%[107/156] of all Y chromosome microdeletions). Third, we reported for the first time the high incidence of Y chromosome microdeletions in the Chinese population with cryptozoospermia, comprising combined deletions of sY254, sY255, and sY152; thus emphasizing the recommendation for Y chromosome microdeletion screening for patients with cryptozoospermia. If the combined deletion of sY254, sY255, and sY152 is detected in patients suspected of azoospermia, sperm should be detected using repeated semen centrifugation to avoid misdiagnosis. Fourth, we provided a reference for comprehending the clinical significance and detection relevance of AZFd deletions. The absence of SY145 in AZFd was closely associated with azoospermia, suggesting this region’s involvement in spermatogenic functions. Fifth, we are the first to explore ICSI outcomes in patients with combined deletion of STS sites in the AZFc and AZFd regions.

This study had some limitations. First, we did not distinguish between non-obstructive and obstructive azoospermia because some of these patients did not undergo ultrasound examination of the genital system. Second, we performed propensity score matching to match the basic characteristics of the AZFcd deletion and control groups. However, this method cannot eliminate potential biases. Third, this study used a small sample size to explore the effect of AZFcd deletions on ICSI outcomes. A larger sample size may be required to further substantiate our findings. Fourth, this research was conducted exclusively within the Chinese population; therefore, its generalizability to other populations may be limited. Furthermore, this study should be replicated in larger cohorts and different populations. Fifth, this study only grouped the enrolled patients based on sperm count, and did not discuss the influence of Y chromosome microdeletions on sperm motility and morphology. Sixth, the EAA/EMQN best practice Guidelines provide a clear flowchart for handling inconsistencies in genetic markers such as AZFa (sY84, sY86), AZFb (sY127, sY134), and AZFc (sY254, sY255) [3]. These guidelines recommend repeating the test in single-plex or adjusting PCR conditions to rule out potential artificial PCR results in cases of marker inconsistency. When both markers are reported as deleted, testing additional markers to confirm the complete deletion in that region is advised. Regrettably, we did not follow these recommendations for cases where only one marker was deleted in the AZFa region, nor did we conduct extension analysis when both markers were missing in an AZF region. These deviations from the EAA/EMQN guidelines may have impacted the accuracy of our study. Going forward, similar studies must adhere to the EAA/EMQN guidelines for reliable and consistent results.

## Conclusions

This study provides the most comprehensive data on Y chromosome microdeletion among Chinese men with infertility, revealing an incidence of 3.31% among men with infertility. Our study revealed a high detection rate of Y chromosome microdeletions in Chinese men with infertility having severe oligozoospermia (total sperm number < 10 × 10^6^ per ejaculate), cryptozoospermia, and azoospermia. This suggests that Y chromosome microdeletion testing should be considered when the total sperm numbers are below 10 × 10^6^ per ejaculate. The absence of SY145 in AZFd is closely associated with azoospermia, suggesting that this region may be involved in spermatogenic functions. The most prevalent pattern of Y chromosome microdeletions among Chinese men with infertility involved the combined deletion of sY254 and sY255 in the AZFc region and sY152 in the AZFd region. For oligozoospermia and cryptozoospermia, patients with AZFcd deletions (combination of sY254, sY255, and sY152 deletions) using ejaculated sperm can achieve ICSI outcomes similar to those in patients without Y chromosome microdeletions. Our study provides an important reference for detecting Y chromosome microdeletions in Chinese men with infertility.

### Electronic supplementary material

Below is the link to the electronic supplementary material.

Additional file 1 Description of data: table with detailed results of semen analysis and Y-chromosome microdeletion analysis of the 4,714 men with infertility.

## Data Availability

The data are available from the corresponding author on reasonable request.

## List of Abbreviations

2PN: Two pronuclei
AZF: Azoospermia factor
BMI: Body mass index
ICSI: Intracytoplasmic sperm injection
STS: Sequence-tagged site
